# 6,7,9,10-Tetra­hydro-16,22-ethano­oxyethano-5,8,11,19-tetra­oxa-16,22-diaza­dibenzo[*h*,*q*]cyclo­octa­decine-17,21-dione: a benzyl­annelated macrobicyclic diamide

**DOI:** 10.1107/S1600536808030067

**Published:** 2008-09-24

**Authors:** Gary L. N. Smith, Yang Lei, Douglas R. Powell, Richard W. Taylor

**Affiliations:** a Department of Chemistry, Point Loma Nazarene University, 3900 Lomaland Dr., Rohr Science 305E, San Diego, CA 92106, USA; b Department of Chemistry and Biochemistry, University of Oklahoma, 620 Parrington Oval, Room 208, Norman, OK 73019-3051, USA

## Abstract

The macrobicyclic title compound, C_24_H_28_N_2_O_7_, has two tertiary diamide bridgehead atoms and is composed of a 12-membered ring (N_2_O_2_ donor set) and two 18-membered rings (N_2_O_4_ donor sets). The solid-state structure shows that each of the amide groups is not coplanar with the adjacent benzene ring and NMR studies indicate that this conformational relationship persists in solution.

## Related literature

For general background, see: Dietrich *et al.* (1969 ▶); Tummler *et al.* (1977 ▶); Niklas *et al.* (2004 ▶); Schickaneder *et al.* (2006 ▶); Lehn (1973 ▶). For related structures, see: Tarnowska *et al.* (2004 ▶); Smith *et al.* (2007 ▶). For the synthesis, see: Dietrich *et al.* (1973 ▶).  For NMR studies, see: Smith *et al.* (2007 ▶); Silverstein & Webster (1998 ▶).
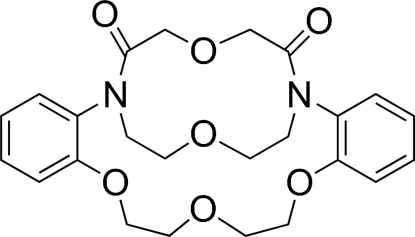


## Experimental

### 

#### Crystal data

C_24_H_28_N_2_O_7_
*M* *_r_* = 456.48
Monoclinic, 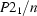

*a* = 15.125 (2) Å
*b* = 9.3901 (14) Å
*c* = 16.446 (2) Å
β = 108.416 (5)°
*V* = 2216.1 (5) Å^3^
*Z* = 4
Mo *K*α radiation
μ = 0.10 mm^−1^
*T* = 87 (2) K
0.58 × 0.56 × 0.52 mm

#### Data collection

Bruker APEX diffractometer
Absorption correction: multi-scan (*SADABS*; Sheldrick, 2007 ▶) *T*_min_ = 0.940, *T*_max_ = 0.950
23465 measured reflections
4352 independent reflections
4142 reflections with *I* > 2σ(*I*)
*R*_int_ = 0.020

#### Refinement

*R*[*F* ^2^ > 2σ(*F* ^2^)] = 0.032
*wR*(*F* ^2^) = 0.085
*S* = 1.03
4352 reflections
298 parameters
H-atom parameters constrained
Δρ_max_ = 0.24 e Å^−3^
Δρ_min_ = −0.23 e Å^−3^

### 

Data collection: *SMART* (Bruker, 1998 ▶); cell refinement: *SAINT* (Bruker, 1998 ▶); data reduction: *SAINT*; program(s) used to solve structure: *SHELXTL* (Sheldrick, 2008 ▶); program(s) used to refine structure: *SHELXTL*; molecular graphics: *SHELXTL*; software used to prepare material for publication: *SHELXTL*.

## Supplementary Material

Crystal structure: contains datablocks I, global. DOI: 10.1107/S1600536808030067/pk2117sup1.cif

Structure factors: contains datablocks I. DOI: 10.1107/S1600536808030067/pk2117Isup2.hkl

Additional supplementary materials:  crystallographic information; 3D view; checkCIF report

## Figures and Tables

**Fig. 1.**
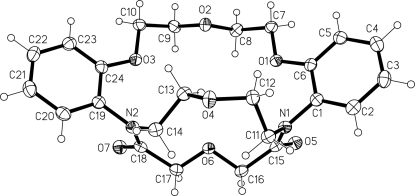
The molecular structure of (I), showing the atom-numbering scheme. Displacement ellipsoids are drawn at the 50% probability level.

## supplementary crystallographic information

### Comment

Cryptands (Dietrich *et al.*, 1969; Lehn, 1973) and tertiary amides (Tummler *et al.*, 1977; Niklas *et al.*, 2004; Schickaneder *et al.*, 2006) are of interest as hosts for cationic guests. The title compound, (I), was isolated during the synthesis of the corresponding benzoannelated cryptand. A related macrobicyclic diamide without benzene rings has been reported, but the carbonyl groups are on the bridge containing the three ether O atoms (Tarnowska *et al.*, 2004).

Fig. 1 shows that (I) consists of a 12-membered ring (N1, O4, N2, O6) and two 18-membered rings (N1, O1, O2, O3, N2, (O4 or O6)). With respect to the molecular cavity formed by these rings, donor atoms O1, O2, O3, O6, N1, and N2 have an endodentate orientation while O4 and carbonyl oxygen atoms O5 and O7 are exodentate. The donor atoms shared by the 18-membered rings (N1, O1, O2, O3, N2) form a plane (average deviation = 0.0244 Å) that is almost perpendicular (dihedral angle = 92.8 (2)°) to the plane defined by the donor atoms from the 12-membered ring (N1, O4, N2, O6; average deviation = 0.0995 Å). The planar amide groups (N1, C15, O5, C16; average deviation = 0.0014 Å), (N2, C18, O7, C17; average deviation = 0.0060 Å) form dihedral angles of 86.4 (2) and 99.0 (2)° with benzene rings 1 and 2, respectively.

In this conformation the distances between protons H2 and H20 and the carbonyl O atoms (O5 and O7) are 3.70Å and 3.80 Å, respectively. In the solid-state structure of the analogous monocyclic diamide (*i.e.*, donor atoms N1, O1, O2, O3, N2, O6), each amide group and adjacent benzene ring are nearly co-planar (dihedral angles = 14.3 (2)°, 17.1 (2)°) and the distances between protons analogous to H2 and H20 to the adjacent carbonyl O atoms are between 2.29Å and 2.40Å (Smith *et al.*, 2006). In CDCl_3_, the ^1^H chemical shift values of the aromatic protons of (I) lie in the expected range from 6.96 - 7.25 p.p.m. (Silverstein & Webster, 1998); however, for the corresponding monocyclic diamide, the *ortho* protons are shifted downfield to 8.22 p.p.m. due to deshielding by the adjacent carbonyl O atoms. The X-ray structure and NMR chemical shift data for (I) indicate that the presence of the ethanooxyethano bridging strand prevents the amide and benzene groups from adopting a coplanar conformation both in the solid state and in solution.

### Experimental

Compound (I) was obtained from the reaction of the monocyclic diamine (Smith *et al.*, 2007) (3.8 m*M*) in CH_2_Cl_2_ containing pyridine (15 m*M*) and the 2,2'-oxydiacetyl chloride solution (4.3 m*M*) in CH_2_Cl_2_ under high dilution conditions (Dietrich *et al.*, 1973). The crude diamide was purified by flash column chromatography on silica gel using CH_2_Cl_2_ and MeOH (0–10%) as the eluent. Spectroscopic Analysis: ^1^H-NMR (CDCl_3_, 300 MHz) δ 3.66, 3.83 (m, 4H, NCH_2_*CH_2_*), 3,71, 4.44 (m, 4H, NCH*_2_*), 3.85,3.96 (m, 4H, ArOCH_2_*CH_2_*), 4.17 (m, 4H, ArOCH_2_), 4.39, 4.52 (m,4*H*, C(?O)CH_2_), 6.96 - 7.25 (m, 8H, Ar); ESI-MS: m/*z* = 457.3 (*M* + H^+^) and 479.3 (*M* + Na^+^). Crystals suitable for X-ray crystallography were grown by vapor diffusion of MeOH into a solution of (I) in CH_2_Cl_2_.

### Refinement

H atoms were positioned goemetrically and refined using a riding model with C—H = 0.95Å for aromatic carbons and 0.99Å for methylene carbons.

### Crystal data

**Table:**

C_24_H_28_N_2_O_7_	*F*(000) = 968
*M**_r_* = 456.48	*D*_x_ = 1.368 Mg m^−^^3^
Monoclinic, *P*2_1_/*n*	Mo *K*α radiation, λ = 0.71073 Å
Hall symbol: -P 2yn	Cell parameters from 7313 reflections
*a* = 15.125 (2) Å	θ = 2.6–28.2°
*b* = 9.3901 (14) Å	µ = 0.10 mm^−^^1^
*c* = 16.446 (2) Å	*T* = 87 K
β = 108.416 (5)°	Block, colorless
*V* = 2216.1 (5) Å^3^	0.58 × 0.56 × 0.52 mm
*Z* = 4	

### Data collection

**Table:**

Bruker APEX diffractometer	4352 independent reflections
Radiation source: fine-focus sealed tube	4142 reflections with *I* > 2σ(*I*)
graphite	*R*_int_ = 0.020
Detector resolution: 8.366 pixels mm^-1^	θ_max_ = 26.0°, θ_min_ = 2.2°
ω scans	*h* = −18→18
Absorption correction: multi-scan (SADABS; Sheldrick, 2007)	*k* = −11→11
*T*_min_ = 0.940, *T*_max_ = 0.950	*l* = −20→20
23465 measured reflections	

### Refinement

**Table:**

Refinement on *F*^2^	Primary atom site location: structure-invariant direct methods
Least-squares matrix: full	Secondary atom site location: difference Fourier map
*R*[*F*^2^ > 2σ(*F*^2^)] = 0.032	Hydrogen site location: inferred from neighbouring sites
*wR*(*F*^2^) = 0.085	H-atom parameters constrained
*S* = 1.03	*w* = 1/[σ^2^(*F*_o_^2^) + (0.0451*P*)^2^ + 0.7509*P*] where *P* = (*F*_o_^2^ + 2*F*_c_^2^)/3
4352 reflections	(Δ/σ)_max_ = 0.001
298 parameters	Δρ_max_ = 0.24 e Å^−^^3^
0 restraints	Δρ_min_ = −0.23 e Å^−^^3^

### Special details

**Table:**

Geometry. All e.s.d.'s (except the e.s.d. in the dihedral angle between two l.s. planes) are estimated using the full covariance matrix. The cell e.s.d.'s are taken into account individually in the estimation of e.s.d.'s in distances, angles and torsion angles; correlations between e.s.d.'s in cell parameters are only used when they are defined by crystal symmetry. An approximate (isotropic) treatment of cell e.s.d.'s is used for estimating e.s.d.'s involving l.s. planes.
Refinement. Refinement of *F*^2^ against ALL reflections. The weighted *R*-factor *wR* and goodness of fit *S* are based on *F*^2^, conventional *R*-factors *R* are based on *F*, with *F* set to zero for negative *F*^2^. The threshold expression of *F*^2^ > σ(*F*^2^) is used only for calculating *R*-factors(gt) *etc*. and is not relevant to the choice of reflections for refinement. *R*-factors based on *F*^2^ are statistically about twice as large as those based on *F*, and *R*- factors based on ALL data will be even larger.

### Fractional atomic coordinates and isotropic or equivalent isotropic displacement parameters (Å^2^)

**Table:**

	*x*	*y*	*z*	*U*_iso_*/*U*_eq_	
O1	0.64508 (5)	0.74748 (8)	0.62300 (5)	0.02150 (17)	
O2	0.59736 (5)	0.89145 (8)	0.45462 (5)	0.02121 (17)	
O3	0.64605 (5)	0.75428 (8)	0.31729 (5)	0.02270 (17)	
O4	0.57879 (5)	0.34635 (8)	0.45980 (5)	0.02311 (17)	
O5	0.84403 (5)	0.62786 (8)	0.72596 (5)	0.02390 (17)	
O6	0.81395 (5)	0.53553 (8)	0.52618 (5)	0.02129 (17)	
O7	0.85667 (5)	0.62395 (8)	0.34044 (5)	0.02538 (18)	
N1	0.72291 (6)	0.48401 (9)	0.65874 (5)	0.01944 (19)	
N2	0.72987 (6)	0.49648 (9)	0.34218 (6)	0.02010 (19)	
C1	0.66837 (7)	0.54166 (11)	0.70866 (6)	0.0198 (2)	
C2	0.65342 (8)	0.46203 (12)	0.77389 (7)	0.0240 (2)	
H2	0.6820	0.3713	0.7877	0.029*	
C3	0.59674 (8)	0.51404 (12)	0.81940 (7)	0.0265 (2)	
H3	0.5869	0.4593	0.8644	0.032*	
C4	0.55497 (8)	0.64576 (12)	0.79873 (7)	0.0249 (2)	
H4	0.5157	0.6810	0.8292	0.030*	
C5	0.56994 (7)	0.72721 (12)	0.73368 (7)	0.0224 (2)	
H5	0.5413	0.8180	0.7203	0.027*	
C6	0.62683 (7)	0.67631 (11)	0.68798 (6)	0.0193 (2)	
C7	0.60204 (8)	0.88484 (11)	0.60179 (7)	0.0219 (2)	
H7A	0.6167	0.9449	0.6539	0.026*	
H7B	0.5335	0.8740	0.5785	0.026*	
C8	0.63801 (8)	0.95395 (11)	0.53658 (7)	0.0232 (2)	
H8A	0.6234	1.0570	0.5336	0.028*	
H8B	0.7066	0.9433	0.5540	0.028*	
C9	0.63046 (8)	0.96135 (11)	0.39353 (7)	0.0228 (2)	
H9A	0.6994	0.9624	0.4140	0.027*	
H9B	0.6084	1.0612	0.3869	0.027*	
C10	0.59670 (8)	0.88731 (11)	0.30861 (7)	0.0233 (2)	
H10A	0.5288	0.8700	0.2924	0.028*	
H10B	0.6092	0.9465	0.2637	0.028*	
C11	0.68585 (7)	0.35454 (11)	0.60899 (7)	0.0217 (2)	
H11A	0.6858	0.2759	0.6491	0.026*	
H11B	0.7275	0.3269	0.5758	0.026*	
C12	0.58634 (7)	0.37445 (12)	0.54701 (7)	0.0224 (2)	
H12A	0.5437	0.3100	0.5644	0.027*	
H12B	0.5660	0.4735	0.5517	0.027*	
C13	0.61408 (7)	0.45732 (11)	0.41885 (7)	0.0219 (2)	
H13A	0.6441	0.5322	0.4609	0.026*	
H13B	0.5626	0.5012	0.3726	0.026*	
C14	0.68495 (7)	0.39065 (11)	0.38188 (7)	0.0219 (2)	
H14A	0.7333	0.3411	0.4282	0.026*	
H14B	0.6533	0.3187	0.3384	0.026*	
C15	0.80862 (7)	0.53954 (11)	0.67021 (7)	0.0200 (2)	
C16	0.86233 (7)	0.49116 (12)	0.61103 (7)	0.0233 (2)	
H16A	0.9256	0.5330	0.6299	0.028*	
H16B	0.8685	0.3862	0.6131	0.028*	
C17	0.86817 (7)	0.50959 (12)	0.47159 (7)	0.0235 (2)	
H17A	0.8851	0.4074	0.4745	0.028*	
H17B	0.9266	0.5654	0.4919	0.028*	
C18	0.81667 (7)	0.54926 (11)	0.37915 (7)	0.0206 (2)	
C19	0.67883 (7)	0.54321 (11)	0.25679 (7)	0.0203 (2)	
C20	0.67082 (7)	0.45530 (12)	0.18772 (7)	0.0241 (2)	
H20	0.6996	0.3642	0.1965	0.029*	
C21	0.62088 (8)	0.49929 (13)	0.10519 (7)	0.0270 (2)	
H21	0.6153	0.4385	0.0576	0.032*	
C22	0.57953 (8)	0.63187 (13)	0.09301 (7)	0.0262 (2)	
H22	0.5462	0.6627	0.0366	0.031*	
C23	0.58607 (7)	0.72109 (12)	0.16232 (7)	0.0243 (2)	
H23	0.5569	0.8119	0.1531	0.029*	
C24	0.63543 (7)	0.67709 (11)	0.24507 (7)	0.0207 (2)	

### Atomic displacement parameters (Å^2^)

**Table:**

	*U*^11^	*U*^22^	*U*^33^	*U*^12^	*U*^13^	*U*^23^
O1	0.0234 (4)	0.0199 (4)	0.0218 (4)	0.0037 (3)	0.0079 (3)	0.0030 (3)
O2	0.0234 (4)	0.0210 (4)	0.0195 (4)	−0.0021 (3)	0.0071 (3)	−0.0003 (3)
O3	0.0257 (4)	0.0211 (4)	0.0209 (4)	0.0051 (3)	0.0067 (3)	0.0006 (3)
O4	0.0239 (4)	0.0256 (4)	0.0195 (4)	−0.0087 (3)	0.0064 (3)	−0.0022 (3)
O5	0.0218 (4)	0.0226 (4)	0.0245 (4)	−0.0028 (3)	0.0034 (3)	−0.0016 (3)
O6	0.0177 (3)	0.0262 (4)	0.0196 (4)	0.0040 (3)	0.0055 (3)	0.0019 (3)
O7	0.0231 (4)	0.0266 (4)	0.0282 (4)	−0.0026 (3)	0.0105 (3)	0.0011 (3)
N1	0.0182 (4)	0.0184 (4)	0.0203 (4)	−0.0004 (3)	0.0040 (3)	−0.0012 (3)
N2	0.0188 (4)	0.0201 (4)	0.0217 (4)	0.0006 (3)	0.0068 (4)	0.0016 (3)
C1	0.0174 (5)	0.0209 (5)	0.0191 (5)	−0.0032 (4)	0.0030 (4)	−0.0029 (4)
C2	0.0240 (5)	0.0215 (5)	0.0241 (5)	−0.0041 (4)	0.0043 (4)	0.0005 (4)
C3	0.0278 (6)	0.0297 (6)	0.0225 (5)	−0.0089 (5)	0.0086 (4)	0.0004 (4)
C4	0.0210 (5)	0.0315 (6)	0.0229 (5)	−0.0066 (4)	0.0078 (4)	−0.0069 (4)
C5	0.0198 (5)	0.0239 (5)	0.0217 (5)	−0.0005 (4)	0.0037 (4)	−0.0035 (4)
C6	0.0171 (5)	0.0216 (5)	0.0170 (5)	−0.0034 (4)	0.0023 (4)	−0.0015 (4)
C7	0.0250 (5)	0.0183 (5)	0.0207 (5)	0.0037 (4)	0.0047 (4)	−0.0008 (4)
C8	0.0261 (5)	0.0186 (5)	0.0221 (5)	−0.0018 (4)	0.0035 (4)	−0.0014 (4)
C9	0.0248 (5)	0.0195 (5)	0.0271 (6)	0.0012 (4)	0.0127 (4)	0.0026 (4)
C10	0.0248 (5)	0.0221 (5)	0.0246 (5)	0.0062 (4)	0.0101 (4)	0.0043 (4)
C11	0.0231 (5)	0.0181 (5)	0.0220 (5)	−0.0001 (4)	0.0043 (4)	−0.0021 (4)
C12	0.0204 (5)	0.0266 (5)	0.0201 (5)	−0.0034 (4)	0.0062 (4)	−0.0018 (4)
C13	0.0191 (5)	0.0224 (5)	0.0234 (5)	−0.0018 (4)	0.0057 (4)	0.0022 (4)
C14	0.0220 (5)	0.0189 (5)	0.0246 (5)	−0.0016 (4)	0.0071 (4)	0.0013 (4)
C15	0.0186 (5)	0.0191 (5)	0.0196 (5)	0.0021 (4)	0.0022 (4)	0.0041 (4)
C16	0.0174 (5)	0.0291 (6)	0.0210 (5)	0.0028 (4)	0.0026 (4)	0.0025 (4)
C17	0.0179 (5)	0.0282 (6)	0.0245 (5)	0.0034 (4)	0.0070 (4)	0.0001 (4)
C18	0.0194 (5)	0.0193 (5)	0.0245 (5)	0.0024 (4)	0.0087 (4)	−0.0015 (4)
C19	0.0161 (5)	0.0233 (5)	0.0221 (5)	−0.0024 (4)	0.0068 (4)	0.0011 (4)
C20	0.0200 (5)	0.0246 (5)	0.0291 (6)	−0.0017 (4)	0.0098 (4)	−0.0033 (4)
C21	0.0240 (5)	0.0335 (6)	0.0244 (5)	−0.0070 (5)	0.0090 (4)	−0.0072 (5)
C22	0.0224 (5)	0.0350 (6)	0.0202 (5)	−0.0047 (5)	0.0052 (4)	0.0018 (4)
C23	0.0217 (5)	0.0263 (5)	0.0251 (5)	0.0003 (4)	0.0076 (4)	0.0033 (4)
C24	0.0182 (5)	0.0236 (5)	0.0218 (5)	−0.0023 (4)	0.0085 (4)	−0.0003 (4)

### Geometric parameters (Å, °)

**Table:**

O1—C6	1.3606 (13)	C8—H8B	0.9900
O1—C7	1.4373 (12)	C9—C10	1.4979 (15)
O2—C9	1.4173 (12)	C9—H9A	0.9900
O2—C8	1.4197 (12)	C9—H9B	0.9900
O3—C24	1.3570 (13)	C10—H10A	0.9900
O3—C10	1.4390 (12)	C10—H10B	0.9900
O4—C12	1.4270 (12)	C11—C12	1.5395 (14)
O4—C13	1.4323 (13)	C11—H11A	0.9900
O5—C15	1.2268 (13)	C11—H11B	0.9900
O6—C17	1.4151 (12)	C12—H12A	0.9900
O6—C16	1.4181 (12)	C12—H12B	0.9900
O7—C18	1.2274 (13)	C13—C14	1.5238 (15)
N1—C15	1.3540 (14)	C13—H13A	0.9900
N1—C1	1.4407 (13)	C13—H13B	0.9900
N1—C11	1.4738 (13)	C14—H14A	0.9900
N2—C18	1.3544 (14)	C14—H14B	0.9900
N2—C19	1.4408 (13)	C15—C16	1.5211 (15)
N2—C14	1.4683 (13)	C16—H16A	0.9900
C1—C2	1.3835 (15)	C16—H16B	0.9900
C1—C6	1.4050 (15)	C17—C18	1.5195 (15)
C2—C3	1.3925 (16)	C17—H17A	0.9900
C2—H2	0.9500	C17—H17B	0.9900
C3—C4	1.3815 (17)	C19—C20	1.3782 (15)
C3—H3	0.9500	C19—C24	1.4030 (15)
C4—C5	1.3906 (16)	C20—C21	1.3914 (16)
C4—H4	0.9500	C20—H20	0.9500
C5—C6	1.3937 (15)	C21—C22	1.3792 (17)
C5—H5	0.9500	C21—H21	0.9500
C7—C8	1.4953 (15)	C22—C23	1.3927 (16)
C7—H7A	0.9900	C22—H22	0.9500
C7—H7B	0.9900	C23—C24	1.3923 (15)
C8—H8A	0.9900	C23—H23	0.9500
			
C6—O1—C7	116.27 (8)	C12—C11—H11B	109.0
C9—O2—C8	109.73 (8)	H11A—C11—H11B	107.8
C24—O3—C10	117.56 (8)	O4—C12—C11	113.21 (9)
C12—O4—C13	114.50 (8)	O4—C12—H12A	108.9
C17—O6—C16	110.57 (8)	C11—C12—H12A	108.9
C15—N1—C1	118.12 (9)	O4—C12—H12B	108.9
C15—N1—C11	125.07 (9)	C11—C12—H12B	108.9
C1—N1—C11	116.13 (8)	H12A—C12—H12B	107.7
C18—N2—C19	118.10 (9)	O4—C13—C14	107.60 (8)
C18—N2—C14	124.55 (9)	O4—C13—H13A	110.2
C19—N2—C14	117.31 (8)	C14—C13—H13A	110.2
C2—C1—C6	120.26 (10)	O4—C13—H13B	110.2
C2—C1—N1	120.14 (10)	C14—C13—H13B	110.2
C6—C1—N1	119.53 (9)	H13A—C13—H13B	108.5
C1—C2—C3	120.42 (10)	N2—C14—C13	112.46 (8)
C1—C2—H2	119.8	N2—C14—H14A	109.1
C3—C2—H2	119.8	C13—C14—H14A	109.1
C4—C3—C2	119.54 (10)	N2—C14—H14B	109.1
C4—C3—H3	120.2	C13—C14—H14B	109.1
C2—C3—H3	120.2	H14A—C14—H14B	107.8
C3—C4—C5	120.58 (10)	O5—C15—N1	122.50 (10)
C3—C4—H4	119.7	O5—C15—C16	119.00 (9)
C5—C4—H4	119.7	N1—C15—C16	118.50 (9)
C4—C5—C6	120.30 (10)	O6—C16—C15	109.15 (8)
C4—C5—H5	119.8	O6—C16—H16A	109.9
C6—C5—H5	119.8	C15—C16—H16A	109.9
O1—C6—C5	124.62 (9)	O6—C16—H16B	109.9
O1—C6—C1	116.48 (9)	C15—C16—H16B	109.9
C5—C6—C1	118.89 (10)	H16A—C16—H16B	108.3
O1—C7—C8	108.90 (9)	O6—C17—C18	112.08 (8)
O1—C7—H7A	109.9	O6—C17—H17A	109.2
C8—C7—H7A	109.9	C18—C17—H17A	109.2
O1—C7—H7B	109.9	O6—C17—H17B	109.2
C8—C7—H7B	109.9	C18—C17—H17B	109.2
H7A—C7—H7B	108.3	H17A—C17—H17B	107.9
O2—C8—C7	110.79 (9)	O7—C18—N2	122.89 (10)
O2—C8—H8A	109.5	O7—C18—C17	118.56 (9)
C7—C8—H8A	109.5	N2—C18—C17	118.51 (9)
O2—C8—H8B	109.5	C20—C19—C24	120.53 (10)
C7—C8—H8B	109.5	C20—C19—N2	120.09 (10)
H8A—C8—H8B	108.1	C24—C19—N2	119.35 (9)
O2—C9—C10	110.69 (9)	C19—C20—C21	120.36 (11)
O2—C9—H9A	109.5	C19—C20—H20	119.8
C10—C9—H9A	109.5	C21—C20—H20	119.8
O2—C9—H9B	109.5	C22—C21—C20	119.42 (10)
C10—C9—H9B	109.5	C22—C21—H21	120.3
H9A—C9—H9B	108.1	C20—C21—H21	120.3
O3—C10—C9	107.28 (8)	C21—C22—C23	120.83 (10)
O3—C10—H10A	110.3	C21—C22—H22	119.6
C9—C10—H10A	110.3	C23—C22—H22	119.6
O3—C10—H10B	110.3	C24—C23—C22	119.93 (11)
C9—C10—H10B	110.3	C24—C23—H23	120.0
H10A—C10—H10B	108.5	C22—C23—H23	120.0
N1—C11—C12	112.85 (8)	O3—C24—C23	125.15 (10)
N1—C11—H11A	109.0	O3—C24—C19	115.95 (9)
C12—C11—H11A	109.0	C23—C24—C19	118.90 (10)
N1—C11—H11B	109.0		
			
C15—N1—C1—C2	−108.91 (11)	C1—N1—C15—O5	6.14 (15)
C11—N1—C1—C2	62.11 (12)	C11—N1—C15—O5	−164.00 (9)
C15—N1—C1—C6	74.13 (12)	C1—N1—C15—C16	−173.30 (9)
C11—N1—C1—C6	−114.85 (10)	C11—N1—C15—C16	16.55 (14)
C6—C1—C2—C3	0.33 (16)	C17—O6—C16—C15	171.97 (8)
N1—C1—C2—C3	−176.61 (9)	O5—C15—C16—O6	−114.72 (10)
C1—C2—C3—C4	0.33 (16)	N1—C15—C16—O6	64.74 (12)
C2—C3—C4—C5	−0.75 (16)	C16—O6—C17—C18	177.25 (9)
C3—C4—C5—C6	0.51 (16)	C19—N2—C18—O7	−7.04 (15)
C7—O1—C6—C5	0.33 (14)	C14—N2—C18—O7	170.73 (10)
C7—O1—C6—C1	179.68 (8)	C19—N2—C18—C17	175.37 (9)
C4—C5—C6—O1	179.49 (9)	C14—N2—C18—C17	−6.86 (15)
C4—C5—C6—C1	0.15 (15)	O6—C17—C18—O7	130.66 (10)
C2—C1—C6—O1	−179.96 (9)	O6—C17—C18—N2	−51.64 (13)
N1—C1—C6—O1	−3.01 (13)	C18—N2—C19—C20	103.13 (12)
C2—C1—C6—C5	−0.57 (15)	C14—N2—C19—C20	−74.81 (12)
N1—C1—C6—C5	176.39 (9)	C18—N2—C19—C24	−78.59 (12)
C6—O1—C7—C8	173.66 (8)	C14—N2—C19—C24	103.47 (11)
C9—O2—C8—C7	178.33 (8)	C24—C19—C20—C21	1.14 (16)
O1—C7—C8—O2	75.03 (10)	N2—C19—C20—C21	179.40 (9)
C8—O2—C9—C10	173.86 (8)	C19—C20—C21—C22	0.11 (16)
C24—O3—C10—C9	−171.74 (8)	C20—C21—C22—C23	−0.92 (16)
O2—C9—C10—O3	−71.12 (10)	C21—C22—C23—C24	0.48 (16)
C15—N1—C11—C12	−133.07 (10)	C10—O3—C24—C23	5.01 (15)
C1—N1—C11—C12	56.61 (12)	C10—O3—C24—C19	−175.35 (9)
C13—O4—C12—C11	−76.49 (11)	C22—C23—C24—O3	−179.61 (10)
N1—C11—C12—O4	122.34 (10)	C22—C23—C24—C19	0.76 (15)
C12—O4—C13—C14	125.90 (9)	C20—C19—C24—O3	178.77 (9)
C18—N2—C14—C13	101.99 (11)	N2—C19—C24—O3	0.50 (13)
C19—N2—C14—C13	−80.22 (11)	C20—C19—C24—C23	−1.56 (15)
O4—C13—C14—N2	−176.83 (8)	N2—C19—C24—C23	−179.83 (9)
